# Medical Miscellanies

**Published:** 1818-02

**Authors:** 


### PART IV.

### MEDICAL MISCELLANIES.

### THE PORTE FEUILLE, No. XI.

OR,

DEPOT FOR DETACHED FACTS AND INTERESTING PHÆNOMENA, OCCURRING IN THE PRACTICE OF THE FRIENDS OF THE MEDICO-CHIRURGICAL JOURNAL.

"——Trahit quodcunque potest
Atque addit acervo——"

*West India Fever and Elephantiasis.*

Extract of a Letter from R. V. Esq. to Dr. JOHNSON, dated St. Thomas's, 28th of September, 1817.—"This island, I am informed by Medical Gentlemen, was very healthy until last year, when the Scamander touched here; it has since been very much afflicted with fever, a great porportion of cases terminating fatally. It commences with a violent circumscribed pain across the forehead, and behind the orbits, with suffusion of the eyes; sickness of the stomach; excessive pain across the loins, and sensation as though the limbs were all severely contused. The bowels are sometimes torpid, but generally relaxed. In some cases, hæmorrhages per anum occur, and these are invariably fatal. Mr. Greig, the principal surgeon here, informs me, that any fixed rules for the treatment of the fever would, in nine cases out of ten, be useless—perhaps worse than useless! The features of the disease vary so remarkably and so essentially, that the disease is scarcely alike in any two cases. When first it made its appearance bleeding, *ad deliquium*, in the upright posture, and carried the length of inducing a long faint, invariably left the patient free from all complaint, without any other treatment whatever. The fever, however, will not now be coerced in this manner; nor will it bear a measure which was not relinquished, but after extensive experience of its want of success. Where the vascular energy is not completely developed, and evinced by full, strong pulse, venæsection proves injurious, even in the very first stages of the disease. Still, however, where the pulse evinces strength and hardness, and provided it does not number more than 110 pulsations in the minute, bleeding may be ventured on, at the very onset of the fever. Where the strokes exceed the above number, venæsection is highly prejudicial. In such melancholy cases, mercury shares the same fate with blood-letting—it will not succeed, whatever be the dose or mode of administration. It does not appear that in those who recover, the liver or any organ essential to life, is seriously injured in structure; because the convalescence is most rapid, and no hepatic symptoms ensue. Dissection has been rarely resorted to for many reasons.

*P. S.* I was surprized to see Elephantiasis so common a disease as it here is. It is not uncommon to meet twenty or thirty in the streets, during an hour's perambulation, with feet and legs bearing the most exact similitude to those of an elephant, excepting that they are larger, and covered with a rougher skin; but the shape of the foot and toes is precisely the same. These miserable-looking objects do not seem to feel any pain from the disease, as they walk about, barefooted, on the rough pavement without any apparent difficulty. Those affected appear to belong to the lowest class of society—such as slaves, old, and past work. I cannot learn that there is any particular treatment which proves serviceable in this disgusting complaint. Some of the patients bind plantain leaves on the feet; but most of them leave the disease to the spontaneous efforts of Nature. It is very indolent, and appears to affect the general health but in a very trifling degree.

"My next Letter will be from the Spanish Main, and I hope, from time to time, to furnish you with some interesting particulars relative to the state of medicine in these remote regions."

---

### *Severe Fracture of the Skull with Loss of Substance in the Brain.*

*By* Emanuel Lazzaretto, M. D. &c.

Mr. Newenham, a midshipman, aged 14, fell from the booms into the main hold of His Majesty's Ship Queen Charlotte, a height of 45 feet. He was instantly carried into the sick berth, in a state of entire insensibility, with blood flowing from the mouth and nose. On examining his head, I perceived a wound about three inches in length over the posterior superior portion of the right parietal bone, with a fracture of the skull, which was depressed nearly an inch into the substance of the brain. A crucial incision being made, I found the fracture extend obliquely both upwards and downwards to a considerable distance. A small triangular portion of bone was removed, as was also a small mass of cerebrum that was attached to it. The elevator was introduced, and the depressed bone raised to its proper level; the scalp was brought together with adhesive straps, &c. As soon as the depressed portion of bone was elevated, the patient opened his eyes, and began to call out loudly for some of his messmates, as though he had awaked from a deep sleep. He was bled to xx ounces, and a brisk cathartic of calomel and jalap exhibited. In three months he returned to duty.

*Queen Charlotte, March* 11, 1817. E. M.

*Sudden Death, with the Appearance on Dissection.*

November 12th, 1817, a female child, aged 5 months, who had enjoyed very good health, (with the exception of a slight bowel complaint, with which she was affected four weeks, and which readily yielded to mild remedies) was every now and then observed to catch or hold her breath; and the day before yesterday died suddenly as if from suffocation.

Examination of the body displayed nothing unsound in either the thoracic or abdominal cavities. A little serum was found in the peritoneal sac, and about a spoonful within the pericardium. Right auricle of the heart full of blood, the left side strongly contracted.

*Brain.* The vessels of the pia mater were but a little distended; very little blood was seen in the sinuses; scarcely any perceptible quantity of serum in the ventricles. The medulla oblongata, (and especially the origins of the eighth pair) were found to be surrounded with a plexus of turgid dark-coloured vessels, and serum was here effused in considerable quantity.

No other traces of disease were found than that state of the medulla oblongata above described. T. S.

---

*A Case of Pneumonia terminating fatally; with the Appearances on Dissection.*

John Cambridge, ætatis 24, complained on the 27th of August, of severe pain in the right side, difficulty of breathing, and other symptoms of Pneumonia; forty ounces of blood were immediately abstracted with considerable relief to the patient, a blister was applied to the side, and a diaphoretic draught prescribed. 28th. Symptoms were aggravated since the preceding night. He was put on the strictest antiphlogistic regimen and a cathartic draught immediately given him, which operated briskly. On the 29th, being worse, I prescribed a scruple of calomel (which produced three motions) and a diaphoretic at bed-time. 30th. Febrile symptoms increased; the bleeding was repeated, ad deliquium animi, with considerable relief to every symptom, and a powder of calomel and jalap was given every four hours. *Vespere.* The purgatives operated briskly, but there is some heat of skin. Took a scruple of calomel. 31st. Felt relieved. He took some salts, but they produced no motion. *Meridie.* Every symptom being aggravated, bleeding was again had recourse to with the same relief, and a scruple dose of calomel given. *Vespere.* Pain in the side continues; a blister to be applied, and the calomel repeated. *September 1st.* Calomel produced three motions; side somewhat easier; tongue dry; pulse 96. He took 10 grains of Dover's powder every three hours, and had a blister applied between the shoulders: at mid-day he became much worse; venæsection was repeated to the amount of thirty-two ounces, in consequence of which he felt relieved. At 3, P. M. the usual symptoms were much increased in violence, with subsultus tendinum and singultus, when I ordered him the following draught. ℞. Pulv. ipec. com. gr. x. Ammon. carb. gr. x. Aquæ ℥iſs—part of which only was swallowed. From this time he became gradually worse till five o'clock, when he expired.

*Sectio Cadaveris.* On opening the thorax, about a pint of serum was found in the right cavity; the pericardium itself was healthy, but contained nearly the same quantity of bloody serum; several large polypi of coagulable lymph were found in the ventricles of the heart, which organ was in other respects sound. The lungs adhered firmly to the pleura costalis, and were in a highly inflamed state throughout; the bronchial cells were filled with frothy mucus. On dissecting the right lung, an oblong chalky concretion, about the size of a nutmeg, was discovered, situated high up in the bronchia, which weighed ten drachms. The abdominal viscera appeared perfectly sound.

---

### *Tubercular Eruption from taking cold Drink when the Body was heated.*

Frederick Anterbar, a ship's steward, ætat. 36, admitted into Guy's Hospital January 29th, reports, that on leaving Jamaica seven months ago, he had no appearance of his present complaint. The evening before embarking, being very thirsty after a long walk, he drank about a pint of cold Madeira and water, at a time when he was perspiring profusely. A few minutes after this, he was seized with vertigo; and on attempting to walk, with violent vomiting; attended with great prostration of strength, which has continued, though in a more moderate degree, ever since. On the second day after coming on board, he perceived some small tubercles under the left eye, similar to those now on the hands, and particularly those on the left wrist. He still felt unwell, with a sense of weight over his body, and dizziness in the head and imperfection of vision. The tubercles under the eye continued to enlarge, then began to appear on the forehead, whence they spread down along the nose, and left side of the face. They have not occupied the latter situation more than five weeks—the hands about two months. The general health at present, not bad. Nitrous acid internally, and the warm bath externally, with various remedies directed to the amelioration of the chylo-poetic functions were employed; but with little success.

S.

---

### *Power of Opium in subduing Delirium.*

A prejudice exists against the use of opium in fevers; and this prejudice is well founded, if this powerful substance is used in the first stage, or even in the second, *when due evacuations have not been premised.* Every body knows, that, in the second stage, though the disease may have been ever so actively treated at first, irritability, restlessness, and delirium, are generally the chief source of the patient's protracted distress. Under such circumstances, opium is highly useful and *safe*. I have found it the best of all remedies, for it throws the patient into a calm and long sleep, from which he generally awakes refreshed and rational. It should never be forgotten, that in the secondary stage of fever, delirium arises oftener from continued watchfulness through several previous nights, than from any other cause; hence it is plain, that the best way of removing it is by an appeal to "tir'd Nature's sweet restorer, balmy sleep!" To produce this happy effect, opium must be given with freedom: I have generally exhibited it in doses of from two to three grains, and it has seldom failed of accomplishing the object desired.

I need hardly say, that its use is contra-indicated when a red or *protruded* eye, and other marks of undue determination to the brain, or of topical congestion there, accompany this stage of the disease. In all other circumstances, I hold it safe, and have found it salutary. R.

November 1817.

---

### *Appearances post Mortem in a severe Case of Dysentery.*

The patient had died on the 24th day of the disease. On opening the abdomen, the omentum was found perfectly diaphanous, in consequence of the complete absorption of its fat.

The convolutions of the small intestines shewed no *remaining* traces of inflammation on their peritoneal coat. It was on the colon and rectum that the disease had committed its chief ravages; it had affected their villous, much more than their external coats.

On laying open these intestines, numerous erosions were found, from which drops of dark-coloured blood oozed in considerable quantity. In other places, there were complete deep ulcerated excavations, about the size of a shilling, with jagged irregular edges, and covered, in their bottom, with a black slough, similar to neglected ulcers on the legs, or like the sloughs after gangrenous inflammation.

At one point, the ulcerative absorption had almost consumed the three coats of the rectum, for the finger easily passed through, and formed a rent. When the gut was gently pulled, the upper part tore away without resistance, leaving a portion adhering to the anus. Every part of the rectum that was not occupied by ulcers, had a purple or livid red colour, and gangrenous smell.

The sigmoid flexure of the colon was much contracted in its diameter, and seemed as if it had been begirt by various circles of different sizes, placed here and there, along its course. Its coats too were thickened, pulpy, and cartilaginous. The other parts of the alimentary canal were nearly natural.

On the inferior edge of the right lobe of the liver, there was a small superficial pustular collection of matter; this, when opened, discharged two drachms of yellow pus. The whole viscus was much enlarged, and had a peculiar white appearance, as if coated over with coagulable lymph.

*P. S.* What will those who deny the propriety of blood-letting at the onset of dysentery, say to this? The above case, and the morbid appearances described, are not rare: I could detail many such; but there would be no end to repetitions. It may haply prove a memento to those who adhere to the opinion of Dr. Wilson and others; and may prove to them, that this fell disease, in its first stages, is always highly and decidedly inflammatory.

R.

---

### MISCELLANIES.

Quicquid agunt Homines.

---

### *To the Editors of the Medico-Chirurgical Journal & Review.*

Suum cuique tribuito.

GENTLEMEN,

An eruptive disease having lately occurred in the family of a near relative of mine, though not at present in practice, my advice was solicited on the occasion. I had never myself seen a case of Pemphigus, but, from the aspect of the vesicles and other circumstances, had very little hesitation in believing it to be this disorder. I was hence led to refer to the descriptions, &c. given of this complaint by different writers; and among others, to a well-written Essay, by Dr. R. Hall, in the third volume of the Edinburgh Annals. In the fourth volume of the same work, the author resumes the subject. After an argumentative and philosophical inquiry into the question of the contagious or non-contagious nature of the disease, and subjecting himself and two other subjects to inoculation with complete impunity, he concludes, from a review of the whole evidence, that the affection in question is merely sporadic, and not communicable by infection. It is not my present purpose to detail the case of my young relative, as it differed in no material circumstance from those recorded by other writers; suffice it to say, that though the family in which it occurred was numerous, and separation not enjoined, yet no individual belonging to it was attacked by the disease. My intention is merely to notice the manner in which the article Pemphigus is written in Thomas's Modern Practice of Physic, the major part of which is copied verbatim from the Essay already mentioned, and the remainder evidently compiled from the same source, without the most distant acknowledgment to the author. In proof of this assertion, I need only entreat your readers to peruse the following passages.

"On the whole, when we comprehensively survey the evidence recorded by recent writers on the subject, as well as that furnished by the present and former cases, we must, I apprehend, be necessarily led to conclude, that the pemphigus major of Sauvages is an affection merely sporadic, and not of a contagious nature. That the symptoms accompanying one or other instances of this affection are those which attend febrile diseases, whether inflammatory or putrid, the cases given by Drs. Dickson and Stewart, and the one recorded by Mr. Christie, sufficiently evince. In practice, therefore, it would appear, the most important distinctions are, to ascertain,—1st. When the fever is of an inflammatory nature, and accompanied with strong and increased action of the vascular system. 2dly. When the fever has a tendency to the typhoid type; is marked by great debility, and symptoms which denote a tendency of the fluids to putrefaction.—In the first case, it will be obvious, that evacuation and other antiphlogistic remedies, suited to the nature of the case, will be proper; as, on the other hand, in the second, it will be equally necessary to shun all evacuations, and to employ those remedies alone which support the strength, and give tone and vigour to the system.

"From the whole concourse of symptoms, in the present, and in the two cases of this affection formerly communicated, we are naturally led to infer, that the disease, in a great measure, depended on a certain state of debility, and a tendency of the fluids to putrefaction.

"The general indications of cure thence deducible are sufficiently obvious.

"In the case now under consideration, on the first accession of the complaint, when the skin was hot and dry, a mild antimonial was exhibited, in order principally to excite a gentle diaphoresis; but its use was soon discontinued. Afterwards, opiates combined with vitriolic æther, were found very useful in diminishing the effects of irritation, and in promoting the determination to the surface. The bark and other tonics, particularly the nitrous acid, in a state of proper dilution, were early administered, and proved very effectual in obviating the effects of debility. By these means, and the ulterior employment of other auxiliaries, the health of the patient was speedily re-established." *Hall's Essay on Pemphigus.—Annals of Medicine*, 1799, p. 336.

"On taking a comprehensive survey of what has been recorded by recent writers on the subject, we must, I think, conclude, that pemphigus is an affection merely sporadic, and not of a contagious nature; and that the symptoms accompanying one or other instances of this affection are those which attend febrile diseases, whether inflammatory or putrid. The most important distinctions necessary to be ascertained appear therefore to be,—1st. Whether the fever is of an inflammatory nature, and accompanied with a strong and increased action of the vascular system; or, 2dly. Whether the fever has a tendency to the typhoid type, and is marked by great debility and other symptoms which denote a tendency of the fluids to putrefaction. It will be obvious, that in the first case, evacuation and other antiphlogistic remedies will be proper; and that in the second, it will, on the contrary, be necessary to shun all evacuations, and to employ those remedies alone which support the strength, and give tone and vigour to the system.

In most cases, the disease seems to be connected with a certain state of debility, and a tendency of the fluids to putrefaction, and therefore the indications of cure are obvious.

Having cleansed the stomach by a gentle emetic, where nausea prevails, and dislodged the contents of the intestines by some mild laxative, such as the saline purgatives, or small doses of calomel, we may then give the Peruvian bark either in infusion, decoction, or powder, along with wine. The mineral acids in a state of proper dilution, if administered early, will likewise be of service in obviating the effects of debility, and any tendency to putrefaction.

On the first accession of the disorder, if the skin is hot and dry, it may be of service to give the saline medicine, with small doses of some mild antimonial, in order to excite a gentle diaphoresis; but these should not be continued long.

To diminish the effects of irritation, opiates combined with vitriolic æther, will be proper.

On recovery, the patient's strength is to be recruited by tonics and other auxiliaries." *—*Thomas's Modern Practice of Physic*, p. 213.

" *Plagiary* has been well defined to be the purloining another man's works, and putting them off as our own." Now, though I am perfectly aware that compilers and dictionary writers of every description, have assumed to themselves great licence in this respect, it must, I think, be admitted to have been rather carried too far in the above instance.

Without entering into the consideration of the advantages or disadvantages of popular medical compilations, I shall only briefly observe, that in order to be useful, they should be the fruit of extensive research, and form a condensed epitome of all that is known on the different subjects on which they treat; but, above all, that their authorities should be *faithfully* and *scrupulously* quoted, in order that the student, who is not content with a kind of second-hand knowledge, may have an opportunity of comparing and judging for himself. Of all species of book-making, I hesitate not to pronounce medical book-making the most mischievous; if the above instance of the manner in which such works are but too often conducted, have a tendency to caution

* I quote from the edition of 1810; but in the last, I understand no alteration has taken place, nor any acknowledgement been made to the author above mentioned.

only one tyro in medicine against relying on such sources of information alone, I shall feel much satisfaction.

Permit me to embrace the present opportunity of calling the respectable part of the profession to a practice I have viewed with astonishment during my short stay in the metropolis, of seeing blazoned on the windows and doors of very many apothecaries' shops "*Variolous Inoculation* GRATIS, &c." If the evidence already before the public, of the power of vaccine inoculation as a preventive of small-pox, be not sufficient to satisfy every candid inquirer, neither would they believe though one arose from the dead to confirm its protective virtue. Whether *ignorance* or *self-interest* be, therefore, the actuating motive for such conduct, I shall not stop to inquire, as, in either case, it becomes the duty of the magistrate, as far as empowered by law, to prevent such lures being held out to the ignorant and unwary, since the mischief is not confined to the individual who is duped by them, but becomes a wide spreading source of infection in the community at large. The baneful habit of suffering children to go abroad in a crowded city while labouring under small-pox, as is the case at present, is, if possible, still more reprehensible, especially as one or two recent convictions show that it *is supineness*, not *want of power*, that prevents an effectual check being opposed to such murderous practices.

I remain, Gentlemen, your constant reader, and obedient servant,

W. SMITH.

London, 25th November, 1817.

---

### *To the Editors of the Medico-Chirurgical Journal & Review.*

GENTLEMEN,

IN surgery, as in medicine, we sometimes meet with cases that can scarcely be made to fall in with the definitions of disease hitherto brought forward by learned and methodical writers.

This consideration, together with some recent observations which the bed-side has afforded, induces me to propose a question which, as the object is purely that of acquiring information, I shall hope some of your numerous and intelligent correspondents will take up.

Dislocations and fractures are occurrences that every practical surgeon is in the habit of occasionally seeing. Upon these subjects, therefore, and upon all those points usually connected with them, he very early in life has an opportunity of making up his mind, from the materials furnished by observation, as well as by reading. There are, however, certain mixed cases, sometimes known to occur, although very rarely; cases in which, from external violence, a partial separation of an epiphysis takes place. The possibility of this description of injury has been generally admitted, but the period assigned for it has been supposed to be that of youth alone, from the circumstance of the union between the epiphyses and bodies of the cylindrical bones being, during that period, still incomplete. I have myself seen several cases of injury occurring near the joints previous to the age of fifteen, which could be referred to this cause only; they were productive of much more pain and tedious confinement than fracture, although connected with scarcely any, if any, obvious displacement of parts.

I have, however, seen, very lately, an instance in which I cannot avoid believing there was a separation of the epiphysis from the lower end of the femur, in a patient forty years of age. She slipped down in the street, and, on attempting to get up, felt convinced, from the peculiar and sudden want of power in the limb, that her "leg was broken." From the peculiar circumstances and appearances of the subsequent displacement, I have not a doubt of the epiphysis having been completely separated. The actual state of the case, however, supposing this to be so, I should not have at last understood, had I not recollected, and profited by, a very remarkable specimen of disease in the femur, which I had examined some years since, in the extensive and brilliant museum of Mr. Heaviside. In this case, the lower part of the thigh bone, evidently from a middle aged person, had, from enlargement and disease, become almost entirely loosened from the condyles, which had not participated in the affection; proving the possibility of separation subsequent to the completion of growth.

I shall feel much obliged by any information upon so curious a subject as the above. It is one but little understood at present, although I have no doubt that some of the ingenious readers of the Medico-Chirurgical Journal will be able to contribute such parallel observations as may at least throw an interesting, if not immediately useful, light upon a point of considerable practical importance.

A CONSTANT READER.

London, December 16, 1817.

---

*To the Editors of the Medico-Chirurgical Journal & Review.*

GENTLEMEN,

IF you think the following case worthy of insertion in your Journal, it is much at your service.

I am yours, faithfully,

THOMAS SEEDS, M.D. Edin.

Portsmouth, Dec. 27, 1817.

J. F. aged 38.—Dec. 5th. Has been subject, for many years, to Rheumatism, especially Lumbago; has had frequent attacks of retention of urine, which, however, readily yielded to fomentations and diuretics, without the use of the catheter. Contracted, about a month since, sores on the prepuce, which got well under seven inunctions of mercurial ointment, by which his mouth was rendered excessively sore, and a profuse ptyalism induced. Was removed to Portsea in an open boat. At present he cannot make water; has lost all power over the left arm, which is very painful to the touch; lower extremities powerless. Pulse strong and hard; feet and legs cold. The catheter was used two or three times only, when he recovered the command of the bladder. Alum gargle; milk diet.

7th. V. S. ad ℥xxvj; hirudines nuchæ necnon emplastrum vesicat. amplum. Legs to be kept warm.

8th. Bore the V. S. well; the pulse, under its influence, became softer and less frequent. A cathartic at night. Can raise the left arm pretty freely.

11th, 12th, and 13th. Tinct. digitalis gt. xx ter die ex aqua. Mouth slowly getting well.

12th and 13th. Warm bath at night, with pulv. Doveri gr. xv. Sinapisms to the feet and loins.

14th. Sweated freely last night; the mustard poultices made him feel very warm: at present he had little remains of his complaint. Mouth nearly well.

Decoct. cinch. ℔j; half diet; liniment. stimul.

From this time he continued to recover rapidly; but complaining of pain and want of power in the *right* shoulder, a blister was applied to the neck, and these symptoms immediately disappeared.

On the 25th of December he was discharged perfectly cured.

The danger of unguarded exposure to cold, while under the influence of mercury, is properly insisted on by practical writers; though, perhaps, such powerful effects from this imprudent exposure would not have occurred in our patient, had he not been so long subject to rheumatism, and thereby predisposed to paralysis.

The older writers on lues venerea speak of epilepsy, mania, apoplexy, palsy, and phthisis, as not uncommon consequences of a course of mercury; we cannot wonder at this, when we reflect, that the great object they proposed to themselves was to induce salivation to as great an extent, and as speedily as possible; and that they estimated the efficacy of the remedy by the quantity of saliva discharged in the day.—Thank God, more rational views of this subject prevail at present.

The pain which this man felt, on the limbs being pressed or handled, is a remarkable feature in his case: I believe this to be peculiar to *rheumatic* paralysis.

In a Negro who laboured under this complaint, and who was last year a patient in Guy's Hospital, the pain was so exquisite on his limbs being touched, that he screamed violently; indeed, he could scarcely endure the pressure of the bed-clothes. This case proved very tedious and untractable; sudorifics, the warm bath, tonics, &c. were all tried in vain. The muscles too wasted prodigiously, and became very flabby; and, if my memory serve me rightly, the pain was the only symptom which yielded. No applications were made to the spine.

Lumbago is well known to be a very painful and obstinate disease, frequently resisting the most powerful sudorifics.—A gentleman of my acquaintance laboured under severe lumbago: a profuse perspiration was induced by sudorifics, without any abatement of the pain of the loins; when, lo! a single application of a sinapism to the small of the back, removed the malady entirely. I have known many analogous examples.

The connection so frequently to be observed between pain in the loins and ischuria, should never be lost sight of; it is equally worthy of note in a practical as in a pathological point of view.

I hold it to be of the first importance in the treatment of nervous diseases (as Dr. Sanders inculcates), to abstract blood pretty freely from the immediate vicinity of the origins of the nerves, whose functions may be impaired or disturbed; following this up by the application of blisters or the use of powerfully stimulant embrocations. Thus we can most readily relieve that local turgescence, or irregular distribution of blood, on which the very essence of nervous diseases depends. By the use of tinct. digitalis, gentle sudorifics, the warm bath, frictions, &c. we equalize the circulation; and by inducing and maintaining general action throughout the system, we prevent irregular determinations of blood to particular parts: of course, scrupulous attention to the state of the digestive organs is indispensably requisite. Where the bowels have been freely opened, but a tendency to constipation still remains, two or three drachms of a neutral salt dissolved in the decoct. cinch. or infus. anthem. serve to keep the bowels soluble, and thus greatly accelerate recovery.

THOMAS SEEDS.

---

### *Pseudo-Mania.*

*Syphilitidem* expellas *Sarsa* tamen usque recurrit.

I perceive that the *Pseudo-Mania* has reached the North, and is making rapid strides in the intellectual city. It has had its day in the *South*, where it threatened to become *epidemic*, but at length a remedy was found in a moderate *mercurial* course. Before the remedy was applied, however, many irreparable damages were done to the constitution; and many a nose has fallen in—many a palate bone has fallen out—and numberless uvulæ have fallen off! I happen to reside in a place where syphilis, I beg pardon, *Pseudo*-syphilis, presents itself on as wide a scale as in any other point in the British dominions, with the exception of the Metropolis, and I have had ample opportunities of marking the effects of the new doctrines, and contrasting the success with that which attended the old fashioned mercurial practice. Now, without any pretensions to the gift of second sight, I most confidently predict, that the Pseudo-syphilitic theory will fall to the ground, "like the baseless fabric of a vision," but—leaving many a "*wreck* behind!"

From long experience, and attention to *venereal* complaints, (for *venereal* we are yet allowed to term them) I am well aware that by rest, evacuations, and mild external applications, *primary* sores will disappear, and even *secondary* symptoms will *seem* to give way. But—"Mark the End;" the enemy, in nine cases out of ten, will return, sooner or later; and, like the fomes of plague—

Viresque acquirit *latendo.*

I have seen enough to convince me of this truth; and I beg to warn the rising generation in the profession, who are so eager to embrace new doctrines, to pause ere they risk their patient's health and their own reputation, by permitting the Pseudo-mania to take possession of their senses. It is well-known to many, that one of the first—I may say the *very first*, surgeon in the British Metropolis, whose talents are inferior to none—whose opportunities of observation are superior to most, expresses himself on this subject in the following terms to his pupils:—"Gentlemen, it is all nonsense—it is all a delusion—nay worse, it is madness!" Without going this length, I may venture to assert, that more mischief is now done by the neglect, than was formerly done by the abuse of mercury in veneral complaints. Some ten or fifteen years back, *secondary* symptoms were infinitely less numerous than they now are, notwithstanding the new light which is said to be thrown on the disease. But what shall we say to the Reports from Edinburgh where so many have been cured without a particle of mercury? Precisely what TIME has said to the Reports from Clifton a few years ago, when *phthisis* was [said to be] cured by hydrogen gas; and to the Reports from all parts of the kingdom respecting the cure of *syphilis* by the *nitrous acid.* Did not primary and secondary symptoms give way, some time ago, to the nitrous acid, as readily as they do now to the decoctum sarsæ, antimonials, and evacuants? But what were the consequences? Why a very short time dispelled the illusion, and verified the prediction then made—

"*Syphilitidem* expellas *acido* tamen usque recurrit."

---

### *New Publications announced.*

Mr. William Cooke, Surgeon-Accoucheur, has announced the publication of an Address to British Females on the Moral Management of Pregnancy and Labour, and some cursory Observations on Medical Deportment; suggested by the death of her Royal Highness Princess Charlotte of Wales; with a Vindication of her Royal Highness's Physicians.

Dr. Charles Parry will speedily publish an Appendix to Dr. Parry's Experimental Inquiry into the Nature of the Arterial Pulse, &c. containing the result of certain experiments announced in that Work.

Small-Pox and Vaccination Hospital, Pancras.—An Account of the number of Patients admitted; also the Out-patients, and the mothers admitted to nurse their Children, from Jan. 1, 1817, to Jan. 1, 1818.

| 1817. | With the casual Small-Pox | | For Ino-culation | | For Vac-cination | | Out-Patients for Vaccination | Mothers |
|---|---|---|---|---|---|---|---|---|
| January | 18 | – | — | – | — | – | 81 | — |
| February | 11 | – | 2 | – | — | – | 168 | 1 |
| March | 7 | – | 7 | – | — | – | 316 | 1 |
| April | 11 | – | 2 | – | — | – | 419 | 1 |
| May | 15 | – | 8 | – | — | – | 366 | 1 |
| June | 27 | – | 6 | – | — | – | 445 | — |
| July | 17 | – | 5 | – | — | – | 362 | 3 |
| August | 7 | – | 1 | – | — | – | 268 | — |
| September | 12 | – | 1 | – | — | – | 334 | — |
| October | 15 | – | 4 | – | 2 | – | 179 | 2 |
| November | 14 | – | 1 | – | 1 | – | 121 | — |
| December | 6 | – | 5 | – | — | – | 65 | — |
| | 160 | | 42 | | 3 | | 3124 | 9 |
| Died | 48 | | 1 | | None | | None | None |

Increased numbers with casual Small-pox – – – – 19
Ditto for Inoculation – – – – – 5
Ditto Out-patients for Vaccination – – 811
Decreased ditto In-patients for Vaccination – – – 2

---

*Books just Published.*

Dr. Crichton on the Vapour of Boiling Tar, as a Cure for Consumption.

Dr. Gordon's Engravings of the Human Skeleton, 8vo.

Dr. Vetch on the Non-contagious Nature of Yellow Fever.

Mr. Foster on Pestilential Fever.

---

Deaths.—In Union Place, Clapham, aged 55, John Perkins Hill, M. D. At Barrackpore (East Indies), aged 61, Dr. James Campbell, Apothecary General.—In the New Road, Somers Town, Mr. Moss, Surgeon.

